# Phenotypic and Genomic Analysis of Antimicrobial Resistance in *Escherichia coli* Isolated from Food-Transport Containers Used in Institutional Catering

**DOI:** 10.3390/antibiotics15040358

**Published:** 2026-03-30

**Authors:** Levente Hunor Husz, Gergely Álmos Tornyos, Eszter Kaszab, Enikő Fehér, András Bittsánszky, András József Tóth, Miklós Süth, Ákos Jerzsele, Ádám Kerek

**Affiliations:** 1 Department of Pharmacology and Toxicology, University of Veterinary Medicine Budapest, István utca 2, H-1078 Budapest, Hungary; husz.levente.hunor@student.univet.hu (L.H.H.); tornyos.gergely.almos@student.univet.hu (G.Á.T.);; 2 National Laboratory of Infectious Animal Diseases, Antimicrobial Resistance, Veterinary Public Health and Food Chain Safety, University of Veterinary Medicine Budapest, István utca 2, H-1078 Budapest, Hungary; kaszab.eszter@univet.hu (E.K.); feher.eniko@univet.hu (E.F.); suth.miklos@univet.hu (M.S.); 3 National Laboratory of Virology, Szentágothai Research Centre, University of Pécs, H-7624 Pécs, Hungary; 4 Department of Microbiology and Infectious Diseases, University of Veterinary Medicine Budapest, István u 2, H-1078 Budapest, Hungary; 5 Department of Bioinformatics, One Health Institute, University of Debrecen, Nagyerdei Krt. 98, H-4032 Debrecen, Hungary; 6 Institute of Food Chain Science, Department of Food Hygiene, University of Veterinary Medicine Budapest, István utca 2, H-1078 Budapest, Hungary; bittsanszky.andras@univet.hu (A.B.); toth.andras.jozsef@univet.hu (A.J.T.)

**Keywords:** antimicrobial resistance, *Escherichia coli*, public catering, food-transport containers, minimum inhibitory concentration, whole-genome sequencing, extended-spectrum β-lactamases, resistome, One Health

## Abstract

**Background**: Public catering is an underexplored One Health interface where structurally complex food-transport equipment may sustain reservoirs of antimicrobial-resistant bacteria. We investigated *Escherichia coli* from reusable institutional catering food-transport containers, focusing on a difficult-to-clean pressure-relief/ventilation valve compartment. Our objectives were to quantify phenotypic resistance using applied clinical breakpoints, assess inhibitor-synergy outcomes in ESBL confirmatory testing, and contextualize inhibitor-positive isolates by whole-genome sequencing (WGS). **Methods**: *E. coli* was isolated from containers sourced from 17 institutions and three central kitchens using ISO 16649-2. Minimum inhibitory concentrations (MICs) were determined by broth microdilution. Extended-spectrum β-lactamase (ESBL) confirmatory testing used cefotaxime/ceftazidime ± clavulanate; inhibitor positivity was defined as a ≥3 two-fold MIC decrease in the presence of clavulanate in isolates meeting CLSI screening thresholds. Inhibitor-positive isolates underwent WGS and CARD-based resistome profiling. **Results**: Resistance was most frequent to colistin (10, 10.8%), followed by doxycycline (8, 8.6%), florfenicol (7, 7.5%), enrofloxacin (4, 4.3%), and gentamicin (3, 3.2%). Third-generation cephalosporin resistance by clinical breakpoints was uncommon (cefotaxime: 2, 2.2%; ceftazidime: 1, 1.1%). Inhibitor-positive ESBL confirmatory phenotypes occurred in 30 isolates (32.3%), which were sequenced. WGS identified 45 resistance-associated genes across inhibitor-positive isolates but detected no classical ESBL genes; all carried chromosomal *ampC/ampH* alongside ubiquitous efflux-associated determinants. All WGS isolates belonged to phylogroup A, with serotype O154:H9 (20, 66.7%) and ST5549 (17, 56.7%) predominating. **Conclusions**: Institutional catering food-transport containers can harbor AMR *E. coli*, with colistin as the most frequent resistance phenotype and frequent inhibitor-positive ESBL confirmatory profiles that, in this set, were not explained by classical ESBL gene carriage. Integrating phenotype, WGS resistomics, and lineage structure supports targeted hygiene surveillance and risk-informed One Health monitoring in mass catering systems.

## 1. Introduction

Antimicrobial resistance (AMR) has become one of the most consequential global health challenges of the 21st century, eroding the effectiveness of therapies in both human and veterinary medicine and amplifying the clinical, economic, and societal burden of infectious diseases [1,2,3,4]. Because AMR emergence and dissemination are shaped by interconnected selective pressures and transmission routes across humans, animals, and the environment, One Health frameworks have become central to contemporary AMR governance and to the development of actionable mitigation strategies [5,6,7]. Within this integrated perspective, the food chain functions as a critical interface where microbial populations, antimicrobial residues, and mobile genetic elements can converge, enabling resistant bacteria—and the resistance determinants they carry—to move between ecological compartments and into human exposure pathways [8,9,10].

Institutional catering represents a distinctive, high-throughput segment of the food chain in which large meal volumes are produced, held, transported, and served under tight time constraints. Although hazard analysis and critical control point (HACCP) systems are widely implemented to manage food safety hazards, real-world compliance and the physical complexity of equipment can create persistent niches that are difficult to clean, disinfect, and monitor [11,12,13]. Food-contact and food-adjacent surfaces in commercial kitchen environments can harbor *Escherichia coli* with antibiotic resistance and pathogenic potential, underscoring that surface-associated reservoirs may remain relevant even outside classical animal-production settings [14,15,16]. In mass catering operations, reusable transport vessels—often handled repeatedly across production, transport, and serving steps—may therefore serve as underappreciated reservoirs for resistant bacteria, particularly when structural features include crevices, vents, or valve assemblies that limit disinfectant penetration and facilitate biofilm formation.

*E. coli* is a widely used hygiene indicator and AMR sentinel because it is common, genetically diverse, and capable of persistence and rapid adaptation [17,18]. In food-associated environments, its ability to form biofilms can support prolonged survival on abiotic surfaces and complicate sanitation control [19]. In the AMR context, *E. coli* can acquire and disseminate resistance determinants through mobile genetic elements [8,20], enabling resistome evolution across interconnected One Health compartments [21,22,23].

Among high-priority resistance phenotypes, ESBL-producing *E. coli* compromise third-generation cephalosporins and frequently co-occur with additional resistance traits [24,25]. Colistin is a last-line agent for certain multidrug-resistant Gram-negative infections, and its resistance is of particular One Health concern due to the spread of plasmid-mediated *mcr* genes and their expanding diversity [26,27]. Because phenotypic ESBL screening and confirmatory testing can be confounded by non-ESBL mechanisms—including AmpC-related β-lactamase background [28,29,30] and envelope/efflux architectures—paired phenotypic and genomic analysis is essential to distinguish classical ESBL gene carriage [31] from ESBL-like phenotypic responses [32,33]. WGS further enables resistome profiling, mobility signal screening, and lineage-aware interpretation, supporting risk-oriented surveillance in food systems [34,35].

In food safety and One Health surveillance, genomics also supports lineage-aware risk assessment, including the capacity to contextualize resistance within broader population structures and evolutionary trajectories. Importantly, despite increasing international attention to AMR in food systems, data remain comparatively limited for certain high-impact operational environments, including institutional catering workflows and the specialized equipment used for meal transport. Evidence from the broader food chain—including resistant *E. coli* detected in meats and food-associated settings—supports the plausibility of exposure and dissemination routes relevant to consumers [14,36,37]. In Hungary, targeted investigations of *E. coli* from specific sectors (e.g., clinical poultry isolates) further illustrate the local relevance of AMR monitoring and the value of comparative context [38,39]. However, dedicated studies focusing on mass catering transport equipment and structurally complex components remain scarce, leaving a knowledge gap with direct implications for hygiene management and One Health risk mitigation.

In this study, we address this gap by characterizing *E. coli* isolates recovered from institutional catering food-transport containers, focusing on a structurally challenging and potentially under-monitored niche: the pressure-relief/ventilation valve compartment. Using standardized phenotypic susceptibility testing and ESBL screening in combination with genomic analyses, we aim to (i) define the resistance profiles present in this equipment-associated reservoir, (ii) identify the underlying resistome and mobile genetic element signals relevant to dissemination potential, and (iii) provide mechanistic context for phenotype–genotype concordance and discordance in β-lactam resistance signatures. By integrating food safety and One Health perspectives, our findings are intended to inform environmental AMR surveillance strategies and to highlight practical control points for risk reduction in institutional catering systems.

## 2. Results

### 2.1. Isolate Set and Phenotypic Susceptibility

**Figure 1:**
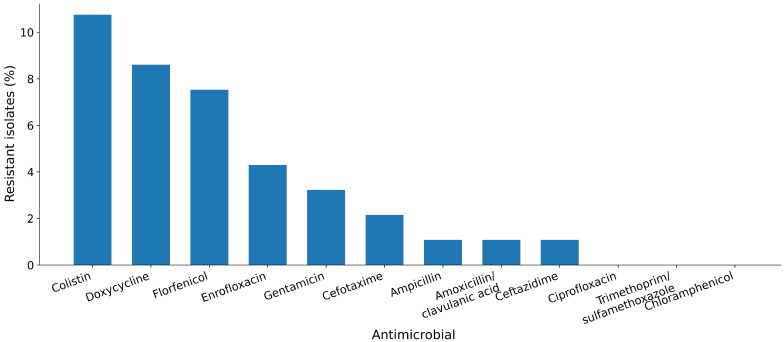
Phenotypic resistance proportions among Escherichia coli isolates from food-transport containers (*n* = 93). Resistant isolates were defined using the applied clinical breakpoints (Table 1).

A total of 93 *E. coli* isolates recovered from institutional catering food-transport containers were tested by broth microdilution against a 16-agent panel (Supplementary Table S1). Resistance was primarily observed for a limited number of antimicrobials (Figure 1). The most frequent resistance phenotype was observed for colistin (10.8%; 95% CI 5.9–18.7%). Resistance was also detected to doxycycline (8.6%), florfenicol (7.5%), enrofloxacin (4.3%), and gentamicin (3.2%). Resistance to third-generation cephalosporins was uncommon by clinical breakpoints (cefotaxime: 2.2%; ceftazidime: 1.1%), consistent with rare classical high-level ESBL-like resistance in the overall isolate set. Only one isolate met the resistant breakpoint for amoxicillin (1.1%), and one isolate met that for amoxicillin/clavulanate (1.1%). MIC_50_ and MIC_90_ values are summarized in Table 1.

### 2.2. Phenotypic ESBL Confirmatory Testing and WGS Isolate Selection

**Figure 2:**
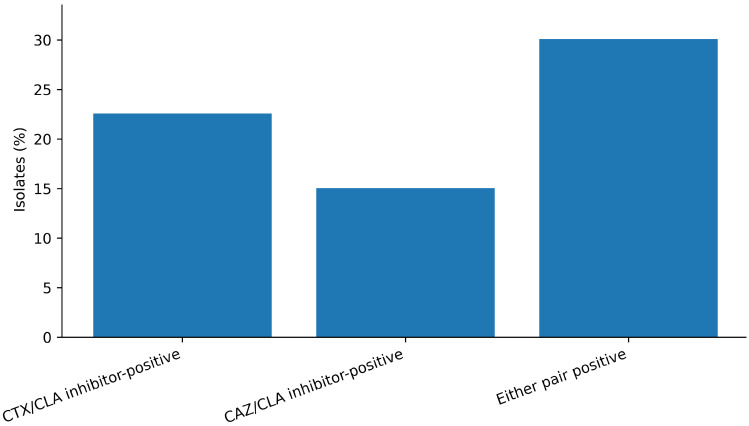
Phenotypic inhibitor-positive extended-spectrum β-lactamase (ESBL) confirmatory outcomes (*n* = 93). Inhibitor positivity was defined as a ≥3 two-fold dilution reduction in minimum inhibitory concentration (MIC) for cefotaxime or ceftazidime in the presence of clavulanate, evaluated in isolates meeting screening thresholds (cefotaxime: CTX ≥ 2 µg/mL; ceftazidime: CAZ ≥ 4 µg/mL).

Phenotypic ESBL confirmatory testing was interpreted using inhibitor synergy criteria (≥3 two-fold dilutions) in isolates meeting the screening thresholds. For cefotaxime, 21 isolates (22.6%) were inhibitor-positive among isolates meeting the screening threshold (CTX MIC ≥ 2 µg/mL). For ceftazidime, 14 isolates (15.1%) were inhibitor-positive among isolates with CAZ MIC ≥ 4 µg/mL (Figure 2; Table 2). Overall, 30 isolates (32.3%; 95% CI 23.6–42.3%) were inhibitor-positive by either confirmatory pair and were selected for WGS. Importantly, screening thresholds (e.g., CTX MIC ≥ 2 µg/mL) are lower than clinical resistance breakpoints (CTX resistant ≥ 4 µg/mL; Table 1); therefore, inhibitor positivity can be frequent even when breakpoint-defined third-generation cephalosporin resistance is uncommon.

### 2.3. Resistome Characterization by WGS in Inhibitor-Positive Isolates

**Figure 3:**
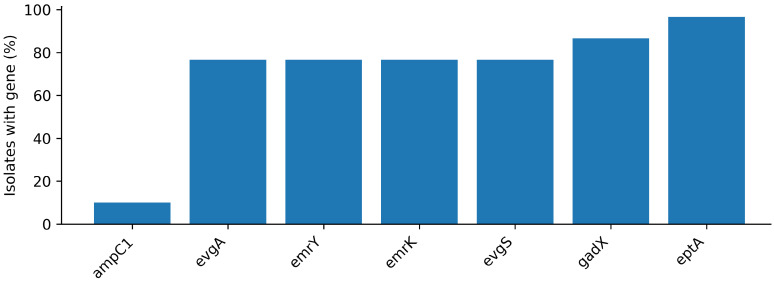
Variable resistance-associated genes across inhibitor-positive whole-genome sequencing (WGS) isolates (*n* = 30). Bars show the proportion of isolates carrying each gene among those with WGS-based resistome profiling.

WGS of the 30 phenotypically inhibitor-positive isolates identified 45 distinct antimicrobial resistance-associated genes (ARGs) across 1290 total detections (Supplementary Table S2). Most isolates carried highly similar ARG repertoires (range 39–45; median 44 genes per isolate), with six genes showing variable presence across the set (Figure 3). No ESBL genes were detected; instead, all isolates carried chromosomal *E. coli ampC* and *ampH* determinants, consistent with a β-lactamase background that may contribute to inhibitor-positive phenotypes. Among the inhibitor-positive isolates subjected to WGS (*n* = 30), the positive predictive value of inhibitor positivity for classical ESBL gene carriage (CTX-M/TEM/SHV-type ESBLs) was 0% (0/30), whereas chromosomal *ampC* and *ampH* were present in 100% of isolates (30/30). Multidrug efflux-associated determinants were ubiquitous across isolates (e.g., *acrAB-tolC* and *mdt* family genes), providing a shared resistance background.

### 2.4. Regulatory Genes and Predicted Genomic Context

**Figure 4:**
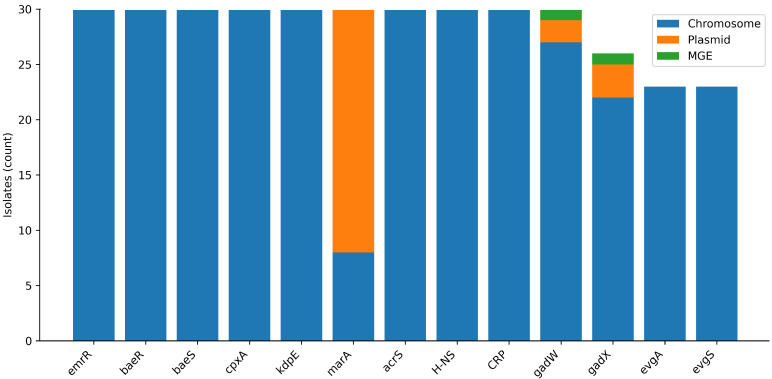
Prevalence and predicted genomic context of selected regulatory genes in inhibitor-positive whole-genome sequencing (WGS) isolates (*n* = 30). Genomic context is reported as chromosome- vs. plasmid-origin contigs predicted by PlasFlow and/or mobile-element association as annotated by MobileElementFinder.

Thirteen resistance- or stress-response-associated regulatory genes were assessed for prevalence and predicted genomic context (Figure 4). Most regulators (*emrR*, *baeR/baeS*, *cpxA*, *kdpE*, *acrS*, *H-NS*, and *CRP*) were present in all isolates and were assigned to chromosomal contigs. Two regulators (*evgA* and *evgS*) were present in 23/30 isolates. Acid stress regulators *gadW* (30/30) and *gadX* (26/30) showed occasional non-chromosomal predictions, including plasmid-origin contigs and, for single isolates, mobile-element association.

### 2.5. In Silico Typing of WGS Isolates

**Figure 5:**
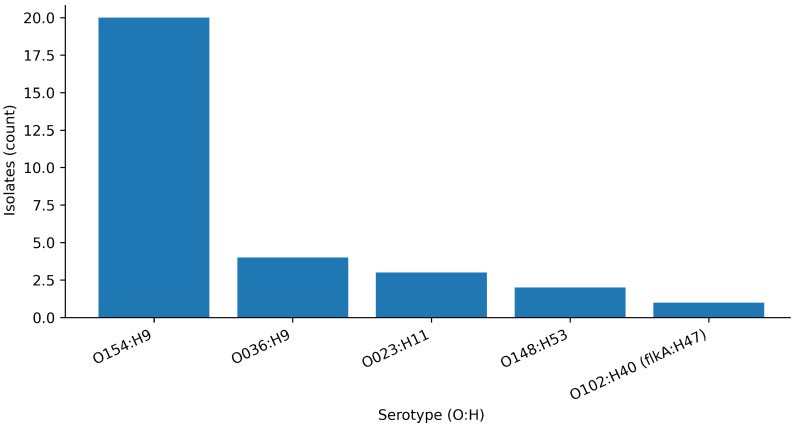
In silico serotype (O:H) distribution among inhibitor-positive whole-genome sequencing (WGS) isolates (*n* = 30).

**Figure 6:**
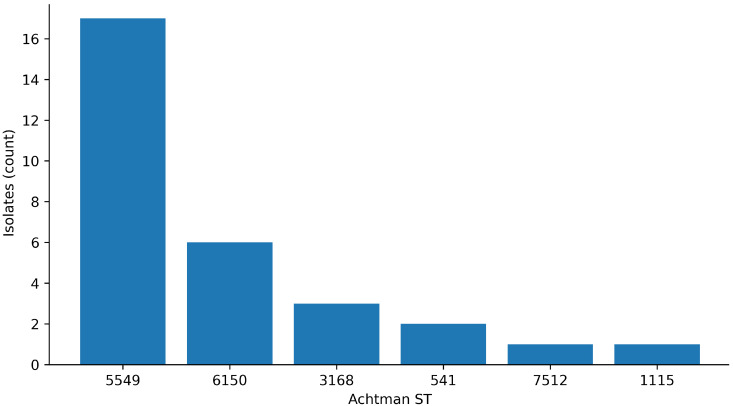
MLST (Achtman) sequence type distribution among inhibitor-positive whole-genome sequencing (WGS) isolates (*n* = 30).

All WGS isolates belonged to phylogroup A (30/30). In silico serotyping identified five O:H serotypes, dominated by O154:H9 (66.7%), followed by O036:H9 (13.3%), O023:H11 (10.0%), O148:H53 (6.7%), and O102:H40 (3.3%) (Figure 5). MLST (Achtman scheme) yielded six STs, with ST5549 predominating (56.7%) (Figure 6). A per-isolate summary linking key MIC values with typing results is provided in Table 3.

### 2.6. Core-Genome Phylogeny and Lineage Structure

**Figure 7:**
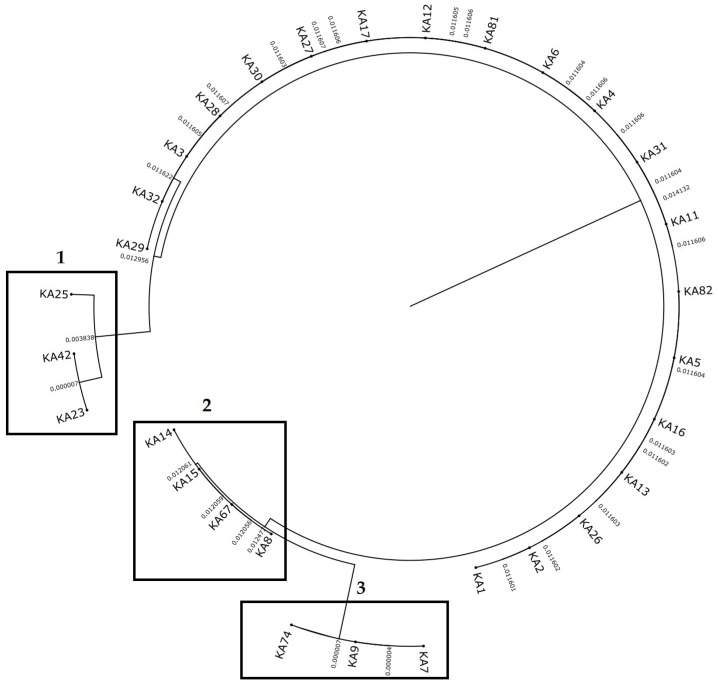
Core-genome phylogeny of inhibitor-positive Escherichia coli isolates from food-transport containers (*n* = 30). The phylogeny is shown with branch lengths as indicated; three short-branch clusters (1–3) are highlighted to improve readability. Tip labels correspond to isolate IDs used throughout (KAxx). Cluster composition: (1) KA23/KA42 with proximal KA25; (2) KA8/KA14/KA15/KA67; (3) KA7/KA9/KA74.

The core-genome phylogeny of the 30 inhibitor-positive isolates indicated limited overall divergence and a dominant background lineage, alongside several short-branch micro-clusters highlighted for readability (Figure 7). Cluster 2 grouped four isolates (KA8, KA14, KA15, KA67) that shared ST6150 and serotype O036:H9, consistent with near-clonal relatedness. Cluster 3 comprised three isolates (KA7, KA9, KA74) assigned to ST3168 and serotype O023:H11. Cluster 1 contained two very closely related isolates (KA23 and KA42; ST541; O148:H53), with KA25 (ST1115; O102:H40) branching proximally. Overall, phylogenetic clustering was broadly concordant with serotype/MLST-defined sublineages within this catering-associated WGS set.

**Figure 8:**
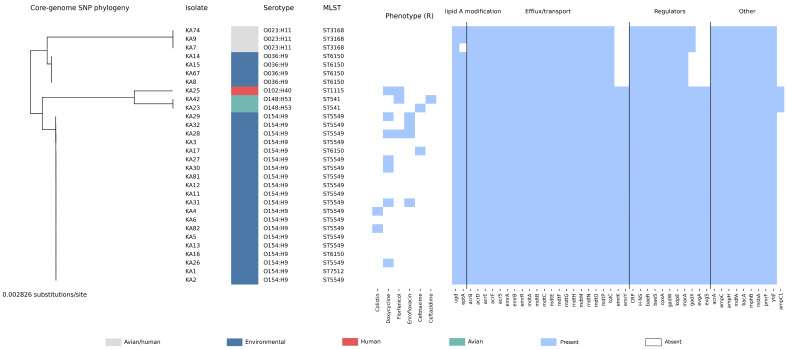
Integrated phylogeny-linked summary of isolate source, typing, phenotype, and resistome in inhibitor-positive whole-genome sequencing (WGS) isolates (*n* = 30). The left panel shows the core-genome SNP phylogeny (branch lengths in substitutions/site) with isolate IDs (KAxx). The colored squares indicate the putative source category for each isolate. Serotype (O:H) and MLST (Achtman ST) are shown as text annotations. The phenotype strip summarizes resistant (R) vs. non-resistant status for selected antimicrobials based on the applied clinical breakpoints. The resistome panel displays presence/absence of selected resistance-associated genes (light blue = present; white = absent), grouped by functional class as indicated (e.g., lipid A modification, efflux/transport, regulators, other).

An integrated phylogeny-linked overview of isolate source, typing, key resistance phenotypes, and selected resistance-associated genes across the WGS subset is shown in Figure 8.

## 3. Discussion

Institutional catering represents a high-throughput, time-pressured segment of the food chain where repeated handling and structurally complex transport vessels can create persistent sanitation blind spots. Here, we provide a lineage-aware, phenotype–genotype integrated snapshot of E. coli associated with a difficult-to-clean, under-monitored component of reusable food-transport containers (the pressure-relief/ventilation valve compartment). Three findings are particularly informative for One Health risk assessment and hygiene management: (i) phenotypic resistance was overall low but non-negligible, with colistin as the most frequent resistance phenotype; (ii) a substantial fraction of isolates displayed inhibitor-positive ESBL confirmatory phenotypes despite the absence of ESBL genes by WGS; and (iii) inhibitor-positive isolates formed a relatively constrained phylogenetic landscape dominated by phylogroup A lineages, with micro-clusters broadly concordant with serotype and MLST sublineages.

Across 93 isolates, resistance (based on applied clinical breakpoints) was concentrated in a limited subset of compounds, suggesting that—at least at the time and sites sampled—this equipment-associated reservoir was not dominated by broadly multidrug-resistant populations. Nevertheless, the observed colistin resistance rate (10/93, 10.8%) is epidemiologically notable because colistin remains a last-line agent for certain multidrug-resistant Gram-negative infections, and its resistance pathways are increasingly recognized as One Health-relevant hazards [24,27]. Although directly comparable data from institutional catering equipment are scarce, colistin resistance and/or *mcr*-associated hazards have been repeatedly documented in food-chain and food-associated *E. coli* across diverse settings, supporting the One Health relevance of this phenotype even when overall resistance remains low [14,37]. Resistance to doxycycline (8/93, 8.6%), florfenicol (7/93, 7.5%), enrofloxacin (4/93, 4.3%), and gentamicin (3/93, 3.2%) indicates that diverse selection histories can converge on this built-environment niche, even when third-generation cephalosporin resistance remains uncommon (cefotaxime 2.2%, ceftazidime 1.1%). Importantly, the ECOFF-based non-wild-type proportions for key cephalosporins were high despite low clinical resistance (e.g., ceftazidime and cefotaxime), suggesting widespread upward MIC shifts that may reflect intrinsic and regulatory architectures (efflux/permeability/β-lactamase background) rather than classical, horizontally acquired ESBL gene carriage. This phenotype distribution is consistent with the concept that built-environment reservoirs can sustain “low-level” resistance signatures that remain below clinical breakpoints yet still matter for selection, persistence, and onward gene-flow potential in food systems [8,9,40].

Comparative context is limited because institutional catering equipment is rarely interrogated at this resolution; however, reports from food-contact surfaces in commercial kitchens and food-related settings have documented antibiotic-resistant *E. coli*, including ESBL-positive and fluoroquinolone-resistant lineages, supporting the plausibility of catering-associated surface reservoirs as AMR interfaces [14,37]. In Hungary, phenotypic AMR profiling in animal-derived clinical *E. coli* isolates has demonstrated that resistance patterns relevant to human medicine circulate locally, motivating surveillance expansion into additional transmission-prone interfaces such as mass catering logistics [38]. Our data refine this picture by showing that even when classical high-risk β-lactam resistance appears rare by breakpoint criteria, other clinically salient phenotypes—especially colistin resistance—can be present in equipment-associated reservoirs.

A central observation of this study is the high frequency of inhibitor-positive ESBL confirmatory outcomes (30/93, 32.3%) contrasted with the absence of classical ESBL genes (e.g., *CTX-M*, *TEM*, *SHV*) in the sequenced subset. This phenotype–genotype discordance is not best interpreted as a “failure” of either approach; rather, it is a mechanistic signal that the inhibitor synergy readout can be produced by alternative architectures beyond ESBL carriage.

One plausible explanation, supported by our genomic findings, is that the baseline chromosomal β-lactamase background (*ampC* and *ampH* detected in all sequenced isolates) interacts with envelope permeability and efflux to yield ESBL-like confirmatory patterns. AmpC β-lactamases are well-established contributors to cephalosporin hydrolysis and can complicate phenotypic β-lactamase interpretation [28]. Although clavulanate classically inhibits many ESBLs and is generally ineffective against AmpC enzymes, apparent synergy can still emerge in complex mechanistic combinations—for example when high-level β-lactamase activity co-occurs with altered permeability and/or efflux-mediated reductions in intracellular antibiotic exposure, thereby shifting the apparent MIC in a way that resembles ESBL inhibition in confirmatory formats [30,31]. AmpH—while typically narrower in substrate profile and not a canonical driver of third-generation cephalosporin resistance—illustrates the broader point that *E. coli* β-lactam response is a layered system, and phenotype can reflect network interactions rather than single determinants [41]. These mechanisms are proposed as plausible contributors based on genomic background and known pathways; however, expression-level validation (e.g., RT-qPCR for *ampC* and efflux systems) was beyond the scope of this study.

This is an important practical message for surveillance in food-associated environments. In resource-limited workflows, inhibitor synergy screens may be used as a triage step for “ESBL suspicion.” Our results caution that, at least in this equipment-associated reservoir, a sizable fraction of inhibitor-positive outcomes may not correspond to classical ESBL gene carriage and therefore require genomic or mechanistic confirmation for accurate risk interpretation. This aligns with broader clinical microbiology experience: phenotype-first screening is indispensable, but its specificity can be reduced by AmpC-related and envelope-associated mechanisms [21,34,35,42].

WGS of inhibitor-positive isolates revealed highly similar ARG repertoires (median 44 genes per isolate; range 39–45), with only six genes varying across the set. This constrained diversity, combined with ubiquitous multidrug efflux determinants (e.g., AcrAB–TolC and multiple Mdt-family modules), suggests that the sequenced population is organized around a shared “core” resistance and stress-adaptation backbone. Such a backbone may not translate into high-level clinical resistance in the absence of activating mutations, regulatory shifts, or specific acquired enzymes, but it can support persistence and rapid adaptive responses under intermittent exposures typical of catering environments (residual disinfectants, sublethal stresses, periodic nutrient pulses, temperature cycling).

The regulatory gene inventory reinforces this view. Multiple systems capable of modulating efflux and envelope responses were present across isolates, including *emrR* (negative regulator of the EmrAB pump) [43], the BaeSR two-component system linked to multidrug exporter activation [44], and global regulators such as CRP that can constrain or reshape efflux expression [45]. Stress-response regulators (*gadX*/*gadW*) can also interface with multidrug resistance pathways, including activation of efflux modules under stress [46]. In combination, these regulatory architectures increase the likelihood that relatively modest genetic changes—or persistent sublethal environmental pressures—could unlock phenotypically meaningful shifts without requiring acquisition of new resistance genes. This interpretation is consistent with the broader efflux literature in *E. coli*, where efflux capacity and its regulation play central roles in multi-compound tolerance and can potentiate other mechanisms [45,46]. Notably, the occasional plasmid- or MGE-associated context predicted for some efflux components in the dataset underscores that, even when the dominant backbone is chromosomal, mobility signals can exist and may become important under selection.

Although all sequenced isolates carried determinants linked to aminoglycoside handling (including *acrD* and *kdpE*), only three isolates were phenotypically gentamicin resistant. This pattern is mechanistically coherent: high-level aminoglycoside resistance in Enterobacterales commonly depends on aminoglycoside-modifying enzymes or other strong determinants [47], whereas efflux contributions (e.g., AcrD) more often provide low-to-moderate MIC shifts [48,49]. The KdpD/KdpE system primarily mediates osmotic and potassium homeostasis but can integrate stress signals that indirectly influence resistance behaviors and virulence-associated traits [50]. In a built-environment niche, such systems may confer “preparedness” under fluctuating stress conditions without guaranteeing a resistant phenotype across all isolates.

Enrofloxacin resistance was present in a minority of isolates. Genomically, we detected efflux-associated modules implicated in fluoroquinolone export (e.g., EmrAB–TolC-related architecture and other transporters), yet efflux alone often does not yield high-level fluoroquinolone resistance in the absence of target-site mutations in QRDR regions (e.g., *gyrA*, *parC*) or strong plasmid-mediated quinolone resistance factors [51,52,53]. Thus, the limited phenotypic resistance in our cohort is compatible with a scenario where efflux systems are present but not maximally activated, and/or where QRDR mutations are not broadly enriched in this reservoir. This again underscores the distinction between genetic capacity and expressed phenotype.

The most consequential non-β-lactam signal is colistin resistance. Global literature emphasizes the public-health relevance of plasmid-mediated *mcr* genes and their expanding diversity [26,27], particularly because mobility enables rapid dissemination across reservoirs. In our sequenced inhibitor-positive subset, we did not detect *mcr* genes, supporting a chromosomal, regulatory, or lipid A modification-based explanation for colistin resistance in this setting. Chromosomal pathways involving phosphoethanolamine transferases (e.g., EptA-related systems) and two-component regulation (e.g., PmrAB) are well documented as mechanisms that reduce colistin binding and can confer resistance depending on regulatory state and mutational activation [54,55]. The presence of lipid A modification-associated genes across isolates without uniform phenotypic colistin resistance suggests that expression state, regulatory mutations, or epistatic interactions likely determine whether resistance manifests at the breakpoint level. This is consistent with the broader concept that chromosomal colistin resistance can be conditionally expressed and influenced by regulatory circuitry rather than simply by gene presence [55]. From a One Health perspective, absence of *mcr* reduces immediate concerns about rapid plasmid-mediated spread, but it does not eliminate risk: chromosomal colistin resistance can still seed exposure pathways and, under selection, may facilitate persistence and adaptive trajectories in environmental reservoirs.

All WGS isolates belonged to phylogroup A, and a single serotype/MLST combination dominated the dataset (O154:H9 and ST5549). The core-genome phylogeny further indicated limited overall divergence and identified short-branch micro-clusters aligned with serotype/MLST-defined sublineages. Such structure is consistent with repeated introduction and/or persistence of a limited set of lineages within the sampled catering-associated ecosystem, potentially facilitated by biofilm formation and the protection afforded by complex, hard-to-disinfect micro-niches [19]. That said, inferring directionality of transmission (human-to-environment vs. environment-to-food) is beyond the resolution of this study: without longitudinal sampling, quantitative contamination loads, and matched sampling from hands, food, water, and surrounding surfaces, phylogenetic clustering can be interpreted as “relatedness and persistence potential,” not as proof of a specific transmission route. Accordingly, phylogenetic clustering is interpreted here as relatedness within the sampled set and compatibility with persistence/repeated reintroduction, rather than proof of transmission directionality or longitudinal persistence.

Nevertheless, lineage-aware framing is operationally useful. If a limited number of lineages dominate in particular facilities, targeted interventions (redesign of cleaning protocols for the valve compartment; verification of disassembly and disinfectant penetration; monitoring for recurrence) can be evaluated against whether the same sublineages recur over time. Conversely, a shift toward greater lineage diversity after intervention could indicate reduced persistence of formerly entrenched clones.

Our findings emphasize that institutional catering equipment should be treated as a meaningful AMR surveillance interface rather than a purely “food hygiene” concern. The valve/ventilation compartment appears to function as a plausible persistence niche where *E. coli* lineages with clinically relevant resistance phenotypes—especially colistin resistance—can be maintained. In practice, this supports three applied recommendations:

Hard-to-clean components (valves, vents, gaskets) should be explicitly incorporated into cleaning validation workflows, including disassembly where feasible and verification of disinfectant contact. Routine surface monitoring programs may need to include these micro-niches rather than relying only on easily accessible surfaces.

Inhibitor synergy screens can overestimate classical ESBL carriage in equipment-associated reservoirs enriched for AmpC/envelope mechanisms. When resources permit, genomic confirmation can prevent misclassification and improve risk communication.

Even with low overall resistance, detecting colistin resistance in a food-associated built-environment warrants attention and potentially a focused follow-up, because last-line resistance phenotypes have disproportionate clinical and policy relevance [24,27].

Several limitations should be framed by interpretation. First, WGS was performed on a phenotype-enriched subset (inhibitor-positive isolates), so genomic findings cannot be generalized to the full isolate set without additional sequencing. Second, short-read assemblies and contig-based plasmid/MGE inference can misassign genomic context, particularly for repetitive elements; complementary long-read sequencing would strengthen conclusions regarding mobility and linkage of determinants. Third, we did not quantify gene expression or confirm mechanistic hypotheses experimentally. Given the central phenotype–genotype discordance observed, direct measurement of *ampC* expression (e.g., RT-qPCR) and systematic evaluation of efflux contribution (expression profiling and/or efflux inhibition assays) would be particularly informative [30,31,48,56]. Fourth, because environmental metadata (cleaning schedules, disinfectant types, container age, damage, handling frequency) and matched sampling from potential sources were not integrated, we cannot infer the direction or timing of introductions.

Despite these constraints, the study provides a strong mechanistic and operational foundation. Next steps that would substantially elevate causal resolution include (i) longitudinal sampling before/after targeted sanitation interventions at the valve compartment; (ii) linking isolate lineages to facility-level metadata; (iii) expanded sequencing beyond inhibitor-positive isolates to capture the broader population structure; (iv) QRDR mutation screening for fluoroquinolone-resistant isolates; and (v) functional testing of β-lactamase/efflux interplay underlying inhibitor-positive phenotypes. Together, these additions would enable a transition from descriptive surveillance toward intervention-oriented One Health risk reduction in mass catering systems.

## 4. Materials and Methods

### 4.1. Study Setting, Sampling, and Isolate Collection

Reusable institutional catering food-transport containers were obtained from 17 institutions and three central kitchens between 18 September 2024 and 15 October 2024 In total, 250 containers were sampled, yielding 400 swab samples from the pressure-relief compartment (a structurally complex, difficult-to-clean micro-niche). For each container, the target compartment was accessed aseptically and sampled using a sterile, pre-moistened swab (sterile saline), which was transported to the laboratory under cooled conditions and processed within 2 h.

After selective isolation (ISO 16649-2 [57]), presumptive *E. coli* colonies were confirmed and stored. To minimize clonal over-representation, one confirmed isolate per sampled container was retained for downstream antimicrobial susceptibility testing whenever possible; if multiple morphotypes were present, a single representative colony was selected based on distinct morphology. Thus, the isolate collection represents container-level sampling units rather than within-container diversity. We did not perform within-sample clonal de-duplication by genotyping prior to AST; however, lineage structure was subsequently assessed in the WGS subset by MLST/serotyping and core-genome phylogeny, which provides post hoc evidence regarding relatedness and potential redundancy.

### 4.2. Broth Microdilution Susceptibility Testing and MIC Interpretation

Phenotypic prescreening for whole-genome sequencing (WGS) selection was based on minimal inhibitory concentration (MIC) determination using a broth microdilution approach following CLSI methodology [58]. Clinical breakpoint interpretation was primarily based on CLSI criteria [58]; where CLSI breakpoints were unavailable, EUCAST breakpoints were applied [59]. In addition, results were evaluated against EUCAST epidemiological cut-off values (ECOFFs) to differentiate wild-type from non-wild-type populations. The applied clinical breakpoints and ECOFF values are summarized in Table 1. For each antimicrobial, a single interpretive standard was applied as specified in Table 1 (CLSI where available; EUCAST only when CLSI breakpoints were unavailable), thereby avoiding mixed classification for any given agent.

Isolates stored at −80 °C were subcultured the day before testing in 3 mL cation-adjusted Mueller–Hinton broth (CAMHB) and incubated for 18–24 h at 37 °C. MIC testing was performed in 96-well microtiter plates (VWR International, LLC., Debrecen, Hungary). Working plates were filled with 90 µL CAMHB per well. Antimicrobials (Merck KGaA, Darmstadt, Germany) were prepared as 1024 µg/mL stock solutions according to CLSI guidance. A two-fold serial dilution was generated across columns 1–10 by transferring 90 µL from the first column into subsequent wells containing 90 µL CAMHB, discarding 90 µL after the final dilution step to maintain equal volumes. Bacterial suspensions were adjusted to 0.5 McFarland using a nephelometer (Thermo Fisher Scientific, Budapest, Hungary) and inoculated at 10 µL per well (from column 11 backwards), consistent with CLSI procedures. Plates were incubated at 37 °C for 18–24 h. MIC endpoints were read using a Sensititre™ SWIN™ automated MIC reader (Thermo Fisher Scientific, Budapest, Hungary) together with VIZION software v3.4 (Thermo Fisher Scientific, Budapest, Hungary, 2024). *E. coli* ATCC 25922 was used as the quality control strain.

Non-wild-type (NWT) status was defined as MIC > ECOFF (not ≥), consistent with standard ECOFF interpretation.

### 4.3. Phenotypic ESBL Screening

Phenotypic ESBL screening was performed following CLSI recommendations [58] using cefotaxime (CTX) and cefotaxime/clavulanic acid (CTX/CLA), and ceftazidime (CAZ) and ceftazidime/clavulanic acid (CAZ/CLA). For clavulanate-containing combinations, clavulanic acid was maintained at a fixed concentration of 4 µg/mL across all dilutions. After preparation of the two-fold dilution series with fixed clavulanate, plates were incubated at 37 °C for 18–24 h and interpreted according to CLSI criteria. An isolate was considered inhibitor-positive in the CLSI ESBL confirmatory test if the MIC in the presence of clavulanate decreased by at least three two-fold dilutions compared with the antimicrobial alone. This phenotypic inhibitor-synergy outcome can be produced by non-ESBL mechanisms (e.g., AmpC-related background combined with permeability/efflux effects) and therefore does not constitute proof of classical ESBL gene carriage.

WGS was intentionally performed on the phenotypically inhibitor-positive subset to maximize the probability of capturing β-lactamase- and envelope-associated determinants relevant to the ESBL confirmatory discordance question, given resource constraints and the study’s primary objective of mechanistic interpretation rather than prevalence estimation. We acknowledge that the absence of an inhibitor-negative comparator limits direct genotype–phenotype contrast within this dataset and therefore interpret WGS findings as descriptive for the inhibitor-positive subset.

### 4.4. Genomic DNA Extraction, Library Preparation, and Whole-Genome Sequencing

Genomic DNA was extracted from pure cultures using the Zymo Quick-DNA Fungal/Bacterial Miniprep Kit (Zymo Research, Irvine, CA, USA) according to the manufacturer’s instructions [60]. Mechanical disruption was performed with a Qiagen TissueLyzer LT (Qiagen GmbH, Hilden, Germany) at 50 Hz for 5 min. Extracted DNA was stored at −20 °C until processing.

Paired-end sequencing reads were generated on an Illumina NextSeq 500 platform via Novogene (Beijing, China). The sequencing-by-synthesis principles and paired-end approach follow established descriptions [34,61]. Sequencing libraries were prepared using the Illumina Nextera XT DNA Library Preparation Kit (Illumina, San Diego, CA, USA), with sample-specific indexing using the Nextera XT Index Kit v2 Set A (Illumina, San Diego, CA, USA). Briefly, DNA was diluted to 0.2 ng/µL in a final volume of 2.5 µL. Tagmentation was performed by combining 2.5 µL diluted DNA with 5 µL Tagment DNA buffer and 2.5 µL Amplicon Tagment Mix, followed by incubation at 55 °C for 6 min in an Eppendorf Mastercycler nexus GX2 (Eppendorf SE, Hamburg, Germany) and cooling to 10 °C. Neutralization was performed by adding 2.5 µL Neutralize Tagment buffer and incubating for 5 min at room temperature. Index PCR amplification was carried out using 7.5 µL Nextera PCR Master Mix with 2.5 µL each of i5 and i7 index primers, followed by an initial denaturation (95 °C, 30 s), 12 cycles of amplification (95 °C, 10 s; 55 °C, 30 s; 72 °C, 30 s), and a final extension (72 °C, 5 min), then cooling to 10 °C. Indexed libraries were purified using the Gel/PCR DNA Fragments Extraction kit (Geneaid Biotech, New Taipei City, Taiwan) following the column purification protocol and quantified fluorometrically with the Qubit dsDNA HS Assay kit (Thermo Fisher Scientific, Waltham, MA, USA). Adapter-tagged libraries were diluted to the appropriate concentration and pooled.

### 4.5. Bioinformatic Processing and Genomic Feature Detection

Raw read quality control and preprocessing were performed using FastQC v0.11.9 [62], fastp v0.23.2-3 [63], and Bloocoo v1.0.7 [64] to identify adapter contamination, base composition anomalies, and low-quality regions; TrimGalore v0.6.6 was used for additional filtering and trimming [65]. De novo assembly was performed using MEGAHIT v1.2.9 [66] and SPAdes v4.0.0 [67]. Assemblies were merged using GAM-NGS v1.1b [68] to obtain improved draft genome reconstructions. Assembly quality was assessed using QUAST v5.2 [69] and BUSCO v5 [70]. Genome characteristics were estimated with GenomeScope v2.2 [71]. Open reading frames were predicted with Prodigal v2.6.3 [72].

Antimicrobial resistance genes (ARGs) were identified among predicted ORFs using Resistance Gene Identifier (RGI) v5.1.0 and ABRicate [73] against the Comprehensive Antibiotic Resistance Database (CARD) [74]. Only hits meeting the CARD “STRICT” criteria and showing at least 90% sequence identity and 90% coverage were retained. Potential mobility of identified ARGs was assessed using MobileElementFinder v1.0.3 [75]. ARGs were considered potentially mobile when located within the maximum transposon distance defined for the organism in the MobileElementFinder database. Putative plasmid origin of contigs was evaluated with PlasFlow v1.1 [76]. The ≥90% identity and ≥90% coverage thresholds were applied to prioritize high-confidence calls and reduce spurious matches in draft assemblies, consistent with a conservative, risk-oriented resistome interpretation. Plasmid-origin assignments from short-read assemblies were treated as predictive, contig-level signals for coarse genomic context only and were not used to infer plasmid transmissibility. Results for mobile genetic elements and plasmid-associated contigs were filtered to hits spanning at least 10,000 bp. Species-level confirmation of E. coli was supported using CheckM v1.2.2 [77] and Kraken v1.1.1 [78].

### 4.6. MLST and Serotyping

To contextualize resistome findings with lineage structure, in silico MLST was assigned from draft assemblies using mlst (v2.32.2) against the PubMLST *E. coli* Achtman seven-gene scheme (*adk*, *fumC*, *gyrB*, *icd*, *mdh*, *purA*, *recA*). In silico O:H serotyping was performed using ECTyper (v2.0.0) from assembled genomes (and, where applicable, raw reads). Typing outputs were linked to isolate IDs and used for lineage-aware interpretation and reporting in the Results.

### 4.7. Phylogenomic Reconstruction

All assemblies were annotated using prokka 1.14.6 with lineage-specific settings (--genus *Escherichia* --usegenus), then the core genome was identified with Panaroo 1.6.0 using --clean-mode strict [79]. The phylogenetic relationship of isolates was reconstructed with IQ-TREE 2.4.0 using the core genome alignment as input and automatic model selection turned on (-m MFP). The phylogenetic trees were visualized with FigTree v1.4.4 (http://tree.bio.ed.ac.uk/software/figtree/ (accessed on 27 February 2026)) and further edited in Inkscape v1.4.3 (https://inkscape.org) to improve readability [80].

## 5. Conclusions

This study shows that reusable institutional catering food-transport containers can harbor antimicrobial-resistant *E. coli* in a structurally difficult-to-clean valve/ventilation compartment. Phenotypic resistance was overall low but included a notable colistin-resistant fraction, while third-generation cephalosporin resistance remained rare. A substantial proportion of isolates exhibited ESBL-like inhibitor synergy; however, WGS of inhibitor-positive isolates identified a conserved resistome backbone without classical ESBL genes, consistent with non-ESBL mechanisms (e.g., chromosomal β-lactamase background and envelope/efflux architectures) driving phenotype–genotype discordance. Lineage structure was constrained (phylogroup A dominance with concordant MLST/serotype micro-clusters), supporting persistence and/or repeated re-introduction of a limited set of catering-associated sublineages. Integrating targeted hygiene verification of hard-to-clean components with phenotype–genotype surveillance is warranted to reduce One Health-relevant AMR risks in mass catering systems.

## Figures and Tables

**Table 1:** Phenotypic minimum inhibitory concentration (MIC) summary and interpretive criteria (*n* = 93). For colistin, the ECOFF equals the applied clinical breakpoint (2 µg/mL); consequently, isolates with MIC = 2 µg/mL are classified as resistant by breakpoint criteria but are not counted as non-wild-type when NWT is defined as MIC > ECOFF.

Antimicrobial	MIC Range (µg/mL)	MIC_50_ (µg/mL)	MIC_90_ (µg/mL)	Breakpoint (µg/mL)	Standard	Resistant, *n* (%)	ECOFF (µg/mL)	Non-Wild-Type, *n* (%)
Ciprofloxacin	0.007–0.25	0.015	0.03	≥4	CLSI	0 (0.0)	0.064	3 (3.2)
Enrofloxacin	0.015–2	0.015	0.06	≥2	CLSI	4 (4.3)		
Colistin	0.015–16	0.5	2	≥2	EUCAST	10 (10.8)	2	6 (6.5)
Amoxicillin	2–256	4	8	≥32	CLSI	1 (1.1)	8	2 (2.2)
Amoxicillin/ clavulanate	2–128	4	8	≥32	CLSI	1 (1.1)	-	-
Ceftazidime	0.03–32	1	4	≥16	CLSI	1 (1.1)	0.5	82 (88.2)
Ceftazidime/ clavulanate	0.007–1	0.125	0.125	-	-	-	-	-
Cefotaxime	0.06–32	0.5	2	≥4	CLSI	2 (2.2)	0.25	70 (75.3)
Cefotaxime/ clavulanate	0.015–8	0.125	0.25	-	-	-	-	-
Ertapenem	0.007–0.06	0.015	0.015	-	-	-	-	-
Doxycycline	0.03–16	2	4	≥16	CLSI	8 (8.6)	8	8 (8.6)
Gentamicin	0.06–16	2	4	≥16	CLSI	3 (3.2)	2	23 (24.7)
Florfenicol	0.5–32	4	8	≥16	CLSI	7 (7.5)	-	-
Lincomycin	0.5–512	256	256	-	-	-	-	-
Trimethoprim/ sulfamethoxazole	0.007–0.5	0.06	0.125	≥4	CLSI	0 (0.0)	-	-
Chloramphenicol	4–16	8	8	≥32	CLSI	0 (0.0)	16	0 (0.0)

CLSI—Clinical and Laboratory Standards Institute; EUCAST—European Committee on Antimicrobial Susceptibility Testing. -: Not available. Clinical breakpoints were used to classify resistance, whereas EUCAST ECOFFs were used to identify non-wild-type (NWT) isolates; ECOFF-based NWT status indicates an upward MIC shift relative to the wild-type distribution and is not equivalent to clinical resistance.

**Table 2:** Phenotypic extended-spectrum β-lactamase (ESBL) confirmatory outcomes and whole-genome sequencing (WGS) selection (*n* = 93). CTX: cefotaxime; CAZ: ceftazidime; MIC: minimum inhibitory concentration.

Confirmatory Pair	Screening Threshold	Inhibitor-Positive, *n* (%)
Cefotaxime vs. cefotaxime/clavulanate	CTX MIC ≥ 2 µg/mL	21 (22.6)
Ceftazidime vs. ceftazidime/clavulanate	CAZ MIC ≥ 4 µg/mL	14 (15.1)
Either pair positive	As above	30 (32.3)

**Table 3:** Whole-genome sequencing (WGS) isolate typing and key minimum inhibitory concentration (MIC) values.

ID	Collect Date	Cefotaxime	Cefotaxime/Clavulanate	Ceftazidime	Ceftazidime/Clavulanate	Colistin	Enrofloxacin	Gentamicin	Doxycycline	Florfenicol	Phylogroup	Serotype	ST Achtman	Putative Origin
KA1	18 September 2024	2	0.125	4	0.125	0.5	0.015	4	0.5	8	A	O154:H9	7512	Environment
KA2	18 September 2024	2	0.125	4	0.06	0.25	0.015	2	1	4	A	O154:H9	5549	Environment
KA3	18 September 2024	2	0.06	2	0.06	0.5	0.03	0.25	0.5	4	A	O154:H9	5549	Environment
KA4	18 September 2024	2	0.03	2	0.125	2	0.015	1	1	4	A	O154:H9	5549	Environment
KA5	18 September 2024	2	0.125	2	0.06	0.5	0.125	2	2	4	A	O154:H9	5549	Environment
KA6	18 September 2024	2	0.125	2	0.125	0.5	0.06	4	1	4	A	O154:H9	5549	Environment
KA7	18 September 2024	2	0.125	2	0.06	0.5	0.015	2	0.5	4	A	O023:H11	3168	Avian/human
KA8	18 September 2024	2	0.125	4	0.125	0.5	0.015	1	1	4	A	O036:H9	6150	Environment
KA9	18 September 2024	1	0.06	4	0.125	1	0.015	4	2	8	A	O023:H11	3168	Avian/human
KA11	7 October 2024	2	0.06	4	0.125	0.5	0.015	2	2	4	A	O154:H9	5549	Environment
KA12	7 October 2024	2	0.06	4	0.125	1	0.015	2	2	2	A	O154:H9	5549	Environment
KA13	7 October 2024	0.25	0.06	4	0.125	0.25	0.015	2	2	2	A	O154:H9	5549	Environment
KA14	7 October 2024	0.25	0.06	4	0.125	0.5	0.015	4	1	4	A	O036:H9	6150	Environment
KA15	7 October 2024	0.25	0.06	4	0.125	0.5	0.03	2	1	4	A	O036:H9	6150	Environment
KA16	7 October 2024	0.25	0.06	4	0.125	0.5	0.03	2	1	4	A	O154:H9	6150	Environment
KA17	7 October 2024	32	0.125	2	0.06	1	0.03	1	0.03	4	A	O154:H9	6150	Environment
KA23	8 October 2024	4	0.06	1	0.125	1	0.015	2	1	4	A	O148:H53	541	Avian
KA25	8 October 2024	2	0.125	2	0.125	0.5	0.015	2	16	16	A	O102:H40	1115	Human
KA26	8 October 2024	2	0.125	2	0.125	0.25	0.015	4	16	8	A	O154:H9	5549	Environment
KA27	8 October 2024	2	0.06	2	0.125	0.125	0.015	4	16	8	A	O154:H9	5549	Environment
KA28	8 October 2024	2	0.125	2	0.125	0.125	2	1	16	16	A	O154:H9	5549	Environment
KA29	8 October 2024	2	0.125	2	0.125	0.5	2	2	16	8	A	O154:H9	5549	Environment
KA30	8 October 2024	2	0.125	1	0.125	0.125	0.03	4	16	8	A	O154:H9	5549	Environment
KA31	8 October 2024	2	0.125	1	0.125	0.25	2	2	16	8	A	O154:H9	5549	Environment
KA32	8 October 2024	2	0.125	2	0.125	0.25	2	4	1	8	A	O154:H9	5549	Environment
KA42	8 October 2024	1	0.06	32	0.007	0.25	0.015	1	2	16	A	O148:H53	541	Avian
KA67	14 October 2024	0.5	0.06	4	0.125	0.5	0.015	2	1	4	A	O036:H9	6150	Environment
KA74	15 October 2024	2	0.03	1	0.125	0.5	0.015	2	2	4	A	O023:H11	3168	Avian/human
KA81	15 October 2024	0.125	0.125	4	0.125	0.5	0.015	4	2	4	A	O154:H9	5549	Environment
KA82	15 October 2024	0.125	0.125	4	0.125	16	0.015	2	2	0.5	A	O154:H9	5549	Environment

## Data Availability

The whole-genome sequencing data generated in this study have been deposited in the NCBI BioProject repository under accession PRJNA1435111 (BioSample submission: SUB16052795). The remaining data are available from the corresponding author upon reasonable request.

## Supplementary Materials

The following supporting information can be downloaded at: https://www.mdpi.com/article/10.3390/antibiotics15040358/s1. Table S1: Isolate-level minimal inhibitory concentration (MIC) values for the tested antimicrobial panel (broth microdilution). Table S2: Resistance-associated genes identified in inhibitor-positive whole-genome sequenced *Escherichia coli* isolates.
